# An effective weapon against biofilm consortia and small colony variants of MRSA

**DOI:** 10.22038/ijbms.2020.46384.10712

**Published:** 2020-11

**Authors:** Zulfiqar Ali Mirani, Shaista Urooj, Muhammad Naseem Khan, Abdul Basit Khan, Izhar Ahmed Shaikh, Anila Siddiqui

**Affiliations:** 1 Microbiology-FMRRC-Pakistan Council of Scientific and Industrial Research Laboratories Complex Karachi, Pakistan; 2 Bioscience, Shaheed Zulfiqar Ali Bhutto Institute of Science & Technology Karachi, Pakistan; 3 Pakistan Council of Scientific and Industrial Research-Islamabad, Pakistan

**Keywords:** AgNPs, Biofilms, MRSA, Nano-Particles, SCVs

## Abstract

**Objective(s):**

This study was designed to investigate the effect of AgNPs (10 nm and 30 nm) on different phenotypes of *Staphylococcus* *aureus* biofilm consortia.

**Materials and Methods:**

A total of eighteen biofilm-producing isolates of Methicillin-Resistant *S. aureus* (MRSA) were used in the present study. Tube methods, Congo-red agar method, and scanning electron microscopy (SEM) were used to study biofilm phenotypes. Population analysis assay on a tryptone soya agar (TSA) plate was applied to study the different phenotypes of biofilm consortia. The effect of AgNPs was evaluated by broth dilution assay.

**Results:**

Results showed that biofilm consortia harbour different phenotypes, i.e., planktonic, metabolically inactive cells, and small colony variants (SCVs) or persister cells. The focus of the present study is the effect of AgNPs on biofilm consortia of MRSA, particularly on the SCVs population. Large size AgNPs (30 nm) were unable to diffuse through extracellular matrix material coverings of the biofilm consortia; they were only active against the planktonic population that occupies the outer surface of consortia. The smaller AgNPs (10 nm), on the other hand, were found to diffuse through the matrix material and hence were effective against planktonic as well as metabolically inactive population of consortia. Moreover, 30 nm AgNPs take 6 hr to disperse off and kill planktonic and upper surface indwellers. The 10 nm AgNPs disperse and kill the majority of biofilm indwellers within 20 min.

**Conclusion:**

The present study showed that 10 nm AgNPs can easily penetrate inside the biofilm and are active against all of the indwellers of consortia.

## Introduction

*Staphylococcus aureus* is one of the major pathogens responsible for community and hospital-acquired infections. It has offered serious challenges for the last two decades due to its antibiotic resistance (1, 2). According to the World Health Organization (WHO) recommendations, it is one of the most dangerous antibiotic-resistant pathogens (3). Up till now, different phenotypes of MRSA have been reported, e.g., hospital-acquired MRSA (HA-MRSA), community-acquired MRSA (CA-MRSA), and live-stock associated MRSA (LA-MRSA). HA-MRSA is predominant in clinical set-ups and is comparatively more resistant, whereas CA-MRSA and LA-MRSA prevail in the community and outside the clinical environment (4). Although, the outbreak of CA-MRSA and LA-MRSA is not common like HA-MRSA these isolates harbour a large number of genes encoding resistance to multiple antibiotics important for human health (5). Moreover, the phenotypic characterization of MRSA suggested that it has a tendency to survive and grow under diverse environmental conditions (6). These pathogens adjust well according to environmental conditions (5). They grow as planktonic in favourable conditions and adopt a biofilm lifestyle during unfavourable conditions (6). Coughlan *et al.*, (7) described that a biofilm is formed when planktonic (or free/stand-alone) cells in an aqueous environment adopt a multicellular lifestyle by attachment to, and colonization of, a solid surface. A biofilm is a highly resistant and protected bacterial life-style covered by self-produced extracellular matrix material made-up of DNA, proteins, carbohydrates, lipids, and dead and active bacterial cells (7). The majority of antibacterial agents are unable to penetrate the protective layer and thus are ineffective against biofilm consortia (8). Consequently, biofilms are a major problem in health care set-ups and food industries, as they can contaminate structural and infrastructural surfaces such as conduits in plumbing, medical implants, food processing facilities, water distribution network, and air conditioning systems (8). In the food industry, mixing tanks, vats, and tubing are favourable sites for biofilm indwellers (8). Once the biofilm is developed in a set-up, it contaminates and deteriorates the product (7, 8). Furthermore, biofilm consortia consist of the heterogeneous population, i.e., planktonic cells, metabolically inactive cells, SCVs, and dead cells, and all phenotypes are highly resistant to conventional antibacterial agents, except the planktonic population (9). These highly resistant indwellers are very difficult to get rid of (7, 8, 10). Therefore, various anti-biofilm molecules, e.g., herbal active compounds, chelating agents, peptide antibiotics, antibiotics, synthetic chemical compounds and nanoparticles have been tested to control biofilms in the food and clinical environment. *In vitro* studies have reported that various synthetic compounds, plant extracts, and nanoparticles could be an alternate option to control biofilms (11). Our previous studies have shown that biofilm consortia harbour different phenotypes (12-14). However, metabolically inactive hydrophobic persister cells were found highly adhesive and resistant to antibacterial agents and environmental stress. Moreover, these phenotypes were found to survive for longer periods under unfavourable environment. Recently, it has been reported that AgNPs can effectively prevent the formation of biofilms and kill bacteria in established biofilms. Therefore, in the present study, different size AgNPs have been applied to control and disperse the highly adhesive, hydrophobic, and metabolically inactive population of MRSA biofilm consortia.

## Materials and Methods

***Identification of S. aureus***

During the study, a total of 18 biofilm-producing isolates of MRSA, recovered from different food commodities, have been studied. For isolation and identification of *S. aureus*, growth was monitored on differential and selective media; for example, Mannitol Salt agar (BioM, Durham, NC, USA), Staphchromo agar (Merck, Darmstadt, Germany), Staphylococcus 110 agar (BioM), Baird–Parker agar (Oxoid, Basingstoke, UK), DNase agar (Merck), and Blood agar (Oxoid). Staph Latex Kit (Prolix Latex Agglutination System, Pro-Lab Diagnostics, South Wirral, UK) was used for confirmation

***Phenotypic characterization of slime-producing bacteria***

Biofilm formation was initially confirmed by the Congo-Red agar method as described earlier (14). Briefly, BHI agar (Oxoid) plates containing 50 g/l sucrose and 0.8 g/l Congo-Red were prepared and streaked with strains and incubated aerobically for 24–48 hr at 37 °C. Positive results were indicated by black colonies with a dry crystalline appearance. Weak slime producers usually remained pink, though occasional darkening at the centre of colonies was observed. 

***Biofilm assay***

A qualitative assessment of biofilm formation on glass slides was evaluated as described earlier by Mirani *et al*., (12-14). Briefly, two-inch piece of glass slide was submerged in tryptone soy broth (TSB) (Oxoid-Hampshire, England) (pH 7.0) containing 0.1 ml culture of the subject isolate and incubated at 35 °C for 24 –72 hr. After incubation, glass slides were taken out from broth and washed with phosphate buffer saline (pH 7.0) to remove unbound cells and debris; biofilms were fixed with acetic acid for 15 min, stained with 3% crystal violet and observed under the microscope. 

***Scanning electron microscopy***

Scanning electron microscopy was carried out to analyze the production of extracellular matrix material after exposure to oxacillin. Biofilm slides were divided into 4-mm sections and washed with distilled water to remove the debris and were then negatively stained with 0.02% uranyl acetate for the 30 sec. These 4-mm slide sections showed the presence of biofilm material when examined directly using a GOEL-JEM-1200 EX II Electron Microscope (JEOL, Peabody, MA, USA) (15, 16).

***Bacterial hydrophobicity assay***

The hydrophobicity of strains was evaluated by the microbial adhesion to solvent test as described in the literature (17); it consisted of evaluating the affinity of the cells toward apolar solvents (hexadecane). For the experiment, bacterial cells were harvested by centrifugation at 8500 g for 5 min and re-suspended in 0.01 M potassium phosphate buffer (pH 7.0). This bacterial suspension was mixed with a solvent (hexadecane) in a ratio of 1:6 (v/v) by vortexing for 3 min to make an emulsion. This mixture was then left for 30 min until the separation of two phases. Aqueous phase absorbance was measured (Abs2) and the percentage of adhesion was expressed as % adhesion= (1 −Abs2/ Abs1) × 100.

***Minimum inhibitory concentration (MIC)***

The antibacterial effect of AgNPs was studied by the method described previously (18). Briefly, the subject isolates of MRSA (10^4 ^CFU/mL) inoculated in TSB broth supplemented with different concentrations of AgNPs were incubated at 35 °C for 6 hr. The 1 ml sample was collected from this cocktail after every 10 min and the effects of AgNPs were recorded by population analysis assay. 

***Effect of AgNPs on biofilms***

Biofilms were developed on glass slides in TSB. The non-adhered cells and debris were removed with a pipette and the plate was washed three times using phosphate buffer saline (pH 7.0). The existing biofilms were exposed to different concentrations of (30 µg/ml, 60 µg/ml, and 120 µg/ml) AgNPs incorporated in TSB and incubated at 35 °C in a shaking incubator (KARL KOLAB-D-6072-Dreieich-West Germany) at 500 rpm. The impact of AgNPs was studied by population analysis, spectrophotometry, and SEM.

## Results

**Figure 1:**
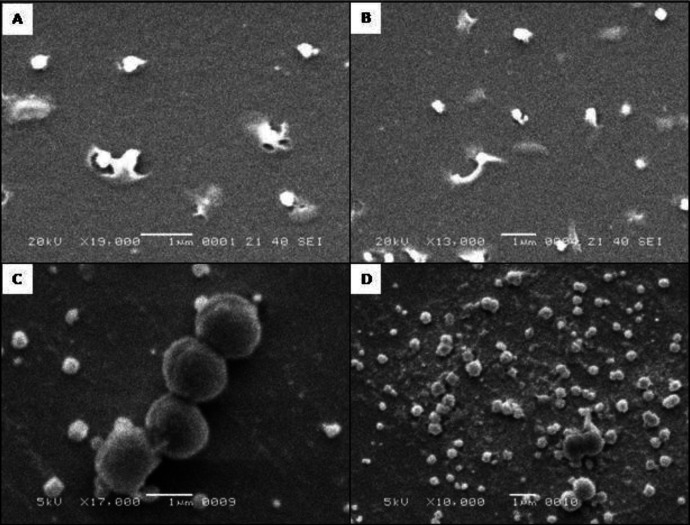
A & B show the lysis of cells of *Staphylococcus arues *after exposure to 10 nm AgNPs. C & D Show the few cells surviving after exposure to 120 µg/ml of AgNPs

Initially, different sizes (10 to 30 nm) of AgNPs were applied on planktonic and biofilm consortia. Investigation of the planktonic population showed that >90% of the cells were killed after exposure to 60 µg/ml of AgNPs within 6 hr (Table 1). The 120 µg/ml of AgNP kills the majority planktonic population in 6 hr, as the smaller size AgNPs (10 nm) showed strong bacterial activity compared to large size particles. This was observed when 120 µg/ml of 10 nm AgNPs was applied against the same strains; it takes < 20 min to kill the majority of the planktonic population. Moreover, biofilm consortia harbour heterogeneous populations and exhibited a heterogeneous type of resistance against AgNPs (Table 1), as after application of highest dose (10 nm) of AgNPs (120 µg/ml), majority of the cells (70 to 100%) were killed or unable to survive and a small minority, i.e., about 25 to 30% of cells continued to exist and proliferate slowly (Figures 1 C & D). The majority of survivors were SCVs and survived even after 6 hr exposure to 120 µg/ml of AgNPs (Table 1). Furthermore, after biofilm formation, the large size AgNPs (30 nm) was ineffective against biofilm indwellers as these large size particles were unable to penetrate inside the matrix martial. However, the AgNPs of 10 nm size could easily penetrate or diffuse inside matrix material and lysed or killed the biofilm indwellers (Figures 1 A & B). The efficiency of AgNPs was found to decrease with biofilm optical density. After 24 hr of incubation, the biofilm optical density was low and the majority of cells were in the planktonic growth phase. The MIC of AgNPs at this stage was low (60 µg/ml) and the majority of the cells were lysed within 20 to 30 min. After 48 hr, biofilm consortia were dominated by metabolically inactive SCVs. The phenotypes were removed by application of 120 µg/ml of 10 nm AgNPs after 6 hr exposure. The other factor that reduces the nano-particle diffusion is the hydrophobic environment of biofilm consortia. The n-hexane binding assay revealed that hydrophobicity of biofilm consortia increases with incubation time and prevalence of SCVs. The study revealed that diffusion of AgNPs inside hydrophobic matrix material is very slow and it takes up to 6 hr to reach the lower base of consortia occupied by SCVs. Moreover, the AgNPs lyse/kill most of the cells in biofilm consortia without damaging the matrix material (Figures 1 A & B). This was confirmed by SEM and biofilm biomass analysis (Table 1). A drastic reduction was noticed in the live population of biofilm consortia after exposure to 10 nm AgNPs, however, the OD assay density showed very minutely or no reduction in biofilm thickness. These results demonstrated that AgNPs are capable to diffuse through the complex network of matrix material but they are unable to break up or dissolve it. 

**Table 1:** Biofilm population analysis of *Staphylococcus arues *before and after application of 10 nm AgNPs (120 µg/ml)

Before AgNP application			After 10 nm AgNP (120 µg/ml) application		
Biofilm OD	Planktonic	SCVs	Biofilm OD	Planktonic	SCVs
0.96	3.3 x10^6^	1.1x10^2^	0.71 (26.04%)	00 (100.0%)	3.1 x10^1^ (26.9%)
0.92	5.1 x106	3.2x103	0.82 (10.87%)	00 (100.0%)	1.2 x10^1^ (69.2%)
0.91	4.7 x10^6^	3.4x10^2^	0.85 (6.59%)	00 (100.0%)	5.2 x10^1^ (32.2%)
0.89	4.9 x10^5^	5.1x10^3^	0.85 (4.49%)	4.3 x10^1^ (71.3%)	5.6 x10^1^ (52.8%)
0.76	3.8 x10^5^	4.2x10^2^	0.71 (6.58%)	3.1 x10^1^ (73.3%)	4.3 x10^1^ (37.7%)
0.73	5.7 x10^6^	6.1x10^3^	0.71 (2.74%)	00 (100.0%)	7.1 x10^1^ (51.1%)
0.89	5.1 x10^6^	7.2x10^3^	0.83 (6.74%)	00 (100.0%)	6.1 x10^1^ (53.7%)
0.89	3.9 x10^5^	7.1x10^3^	0.84 (5.62%)	7.7 x10^1^ (66.3%)	5.2 x10^1^ (55.4%)
0.88	8.3 x10^5^	9.0x10^3^	0.77 (12.50%)	00 (100.0%)	00 (100.0%)
0.86	2.9 x10^5^	3.8x10^2^	0.76 (11.63%)	00 (100.0%)	00 (100.0%)
0.81	8.9 x10^6^	6.9x10^3^	0.76 (6.17%)	3.1 x10^1^ (78.5%)	4.1 x10^1^ (58.0%)
0.81	2.7 x10^5^	5.9x10^2^	0.72 (11.11%)	4.1 x10^1^ (70.3%)	6.1 x10^1^ (35.6%)
0.8	9.7 x10^5^	1.8x10^3^	0.73 (8.75%)	00 (100.0%)	1.2 x10^1^ (66.8%)
0.79	2.5 x10^6^	6.2x10^3^	0.71 (10.13%)	5.1 x10^1^ (73.3%)	4.3 x10^1^ (56.9%)
0.77	3.7 x10^5^	7.1x10^2^	0.71 (7.79%)	00 (100.0%)	00 (100.0%)
0.77	2.8 x10^4^	9.3x10^2^	0.69 (10.39%)	00 (100.0%)	00 (100.0%)
0.76	6.1 x10^6^	9.1x10^3^	0.72 (5.26%)	00 (100.0%)	5.1 x10^1^ (56.9%)
0.75	4.7 x10^6^	2.5x10^2^	0.72 (4.00%)	4.2 x10^1^ (75.7%)	2.6 x10^1^ (41.0%)

SCVs: Small Colony Variants

## Discussion

A total of eighteen biofilms positive MRSA isolates were used in the present study. These isolates adapted biofilm mode of life after 24 hr of incubation and the highest density was achieved at 48 hr of incubation. Population analysis and scanning electron micrographs revealed that this biofilm consortia harbour different phenotypes, i.e., planktonic, metabolically inactive cells and SCVs or persister cells. The SCVs or persister cells occupy the lower base of biofilm consortia and are highly resistant to conventional treatment methods. The metabolically inactive cells occupy the centre of biofilm consortia and planktonic cells are precursors or wild type population that resides on the outer surface of biofilm consortia. The planktonic cells exhibited hydrophilic surface charges, whereas SCVs were hydrophobic. This switching process from planktonic to SCVs and biofilms was under the influence of oxacillin. To overcome this bacterial life-style, different treatment combinations with oxacillin were applied, e.g., ascorbic acid, octadecanoic acid, and silver nanoparticles (12, 13). In our previous studies, octadecanoic acid and ascorbic acid have been shown to have anti-biofilm activity (12, 13). However, both of these compounds were found ineffective against SCVs or metabolically inactive cells (14-16). In the present study, AgNPs were applied against mature or developed biofilms. It has been observed that small size (10 nm) AgNPs could be a good anti-biofilm agent. It easily diffuses through the highly protective biofilm matrix covering and is effective against metabolically active planktonic as well as inactive SCV populations. This is an important finding because SCVs are a highly resistant phenotype, difficult to disperse, and survive for a long time in the adhesive state (15). *S. aureus* is one of the major pathogens that have a tendency to switch life-styles to avoid host defence and antibacterial treatment (17). In a recent study, it has been reported that stressful environment, e.g., reactive oxygen species, low pH, cationic peptides, and limited nutrition, induces development of metabolically inactive SCVs (18). The biofilm environment is a classic example of this, where only the fittest can survive (19, 20). The development of SCVs is a survival strategy of *S. aureus* in biofilm consortia to survive (15). Our previous study indicates that oxacillin induces biofilm mode of life in MRSA and at later stages, the consortia were dominated by SCVs (14, 15). This study showed that the >10nm AgNPs penetrated the extracellular covering of biofilm consortia and effectively killed or dispersed the SCVs. The majority of the available anti-biofilm compounds are trapped in the matrix material and only a few tend to diffuse through it (7). However, these compounds were ineffective against persister cells, metabolically inactive population, and SCVs. A study (21) recommended that smaller size rod shaped NPs are more effective against biofilms due to availability of more surface area for attachment. Smaller NPs have larger specific surface areas, which result in a higher probability of being in touch with and passing through the bacterial cell membrane (22). Another observation of the present study is the increased hydrophobicity of biofilm consortia that results in the prevalence of metabolically inactive cells and SCVs. These phenotypes of MRSA are very difficult to disperse off through conventional treatments. However, the >10 nm AgNPs were found to easily reach and dispersed off the target cells, i.e., SCVs. Researchers (23) described that two hydrophobic surfaces have the strongest interactions due to a local displacement of water molecules. Our study suggested that the diffusion of AgNPs is slow trough the hydrophobic matrix material. However, 10 nm AgNPs slowly diffuse through this protective layer of biofilm consortia and exterminate the highly resistant SCV phenotypes. 

## Conclusion

The observations of the present study suggested that 10 nm AgNPs could be an effective option for the treatment and control of MRSA biofilms. These smaller sized particles are effective against planktonic, metabolically inactive, and hydrophobic populations of the *S. aureus* biofilm consortia. 

## References

[1] Ansari S, Jha RK, Mishra SK, Tiwari BR, Asaad AM (2019). Recent advances in Staphylococcus aureus infection: focus on vaccine development. Infect Drug Resist.

[2] Antri K, Akkou M, Bouchiat C, Bes M, Martins-Simoes P, Dauwalder O, Tristan A, Meugnier H, Rasigade JP, Etienne J, Vandenesch F, Laurent F, Ramdani-Bouguessa N (2018). High levels of Staphylococcus aureus and MRSA carriage in healthy population of algiers revealed by additional enrichment and multisite screening. Eur J Clin Microbiol Infect Dis.

[3] Aslam B, Wang W, Arshad MI, Khurshid M, Muzammil S, Rasool MH, Nisar MA, Alvi RF, Aslam MA, Qamar MU, Salamat MKF, Baloch Z (2018). Antibiotic resistance: a rundown of a global crisis. Infect Drug Resist.

[4] Kateete DP, Namazzi S, Okee M, Okeng A, Baluku H, Musisi NL, Katabazi FA, Joloba ML, Ssentongo R, Najjuka FC (2011). High prevalence of methicillin resistant Staphylococcus aureus in the surgical units of Mulago Hospital in Kampala, Uganda. BMC Res Notes.

[5] Lakhundi S, Zhang K (2018). Methicillin-resistant Staphylococcus aureus: molecular characterization, evolution, and epidemiology. Clin Microbiol Rev.

[6] Kemung HM, Tan LT-H, Khan TM, Chan KG, Pusparajah P, Goh BH, Lee LH (2018). Streptomyces as a prominent resource of future anti-MRSA drugs. Front Microbiol.

[7] Coughlan LM, Cotter PD, Hill C, Álvarez-Ordóñez A (2016). New weapons to fight old enemies: novel strategies for the (bio) control of bacterial biofilms in the food industry. Front Microbiol.

[8] Khatoon Z, McTiernan CD, Suuronen EJ, Mah TF, Alarcon EI (2018). Bacterial biofilm formation on implantable devices and approaches to its treatment and prevention. Heliyon.

[9] Galié S, García-Gutiérrez C, Miguélez EM, Villar CJ, Lombó F (2018). Biofilms in the food industry: Health aspects and control methods. Front Microbiol.

[10] Magana M, Sereti C, Ioannidis A, Mitchell CA, Ball AR, Magiorkinis E, Chatzipanagiotou S, Hamblin MR, Hadjifrangiskou M, Tegos GP (2018). Options and limitations in clinical investigation of bacterial biofilms. Clin Microbiol Rev.

[11] Roy R, Tiwari M, Donelli G, Tiwari V (2018). Strategies for combating bacterial biofilms: a focus on anti-biofilm agents and their mechanisms of action. Virulence.

[12] Mirani ZA, Khan MN, Siddiqui A, Khan F, Aziz M, Naz Sh, Ahmed A, Khan SI (2018). Ascorbic acid augments colony spreading by reducing biofilm formation of methicillin-resistant Staphylococcus aureus. Iran J Basic Med Sci.

[13] Mirani ZA, Naz S, Khan F, Aziz M, Khan MN, Khan SI (2017). Antibacterial fatty acids destabilize hydrophobic and multicellular aggregates of biofilm in Staphylococcus aureus. J Antibiot.

[14] Mirani ZA, Aziz M, Khan MN, Lal I, Hassan N, Khan SI (2013). Biofilm formation and dispersal of Staphylococcus aureus under the influence of oxacillin. Microb Pathog.

[15] Mirani ZA, Aziz M, Khan SI (2015). Small colony variants have a major role in stability and persistence of Staphylococcus aureus biofilms. J Antibiot.

[16] Mirani ZA, Fatima A, Urooj S, Aziz M, Khan M, Abbas T (2018). Relationship of cell surface hydrophobicity with biofilm formation and growth rate: a study on Pseudomonas aeruginosa, Staphylococcus aureus, and Escherichia coli. Iran J Basic Med Sci.

[17] Kouidhi B, Zmantar T, Hentati H, Bakhrouf A (2010). Cell surface hydrophobicity, biofilm formation, adhesives properties and molecular detection of adhesins genes in Staphylococcus aureus associated to dental caries. Microb Pathog.

[18] Akram FE, El-Tayeb T, Abou-Aisha K, El-Azizi M (2016). A combination of silver nanoparticles and visible blue light enhances the antibacterial efficacy of ineffective antibiotics against methicillin-resistant Staphylococcus aureus (MRSA). Ann Clin Microbiol Antimicrob.

[19] Bhattacharya M, Wozniak DJ, Stoodley P, Hall-Stoodley L (2015). Prevention and treatment of Staphylococcus aureus biofilms. Expert Rev Anti Infect Ther.

[20] Trastoy R, Manso T, Fernandez-Garcia L, Blasco L, Ambroa A, Perez Del Molino ML, Bou G, García-Contreras R, Wood TK, Tomas M (2018). Mechanisms of bacterial tolerance and persistence in the gastrointestinal and respiratory environments. Clin Microbiol Rev.

[21] Slomberg DL, Lu Y, Broadnax AD, Hunter RA, Carpenter AW, Schoenfisch MH (2013). Role of size and shape on biofilm eradication for nitric oxide-releasing silica nanoparticles. ACS Appl Mater Interfaces.

[22] Gurunathan S, Han JW, Dayem AA, Eppakayala V, Kim JH (2012). Oxidative stress-mediated antibacterial activity of graphene oxide and reduced graphene oxide in Pseudomonas aeruginosa. Int J Nanomedicine.

[23] Desmau M, Gélabert A, Levard C, Ona-Nguema G, Vidal V, Stubbs JE, Eng PJ, Benedetti MF (2018). Silver nanoparticles dynamics at the solution/biofilm/mineral interface. Environ Sci Nano.

